# Adult cancer risk in women who were breastfed as infants: large UK prospective study

**DOI:** 10.1007/s10654-019-00528-z

**Published:** 2019-06-11

**Authors:** TienYu Owen Yang, Benjamin J. Cairns, Jane Green, Gillian K. Reeves, Sarah Floud, Kathryn E. Bradbury, Valerie Beral, for the Million Women Study Collaborators, Hayley Abbiss, Simon Abbott, Rupert Alison, Miranda Armstrong, Krys Baker, Angela Balkwill, Isobel Barnes, Valerie Beral, Judith Black, Roger Blanks, Kathryn Bradbury, Anna Brown, Benjamin Cairns, Andrew Chadwick, Dave Ewart, Sarah Floud, Kezia Gaitskell, Toral Gathani, Laura Gerrard, Adrian Goodill, Jane Green, Lynden Guiver, Alicia Heath, Darren Hogg, Isobel Lingard, Sau Wan Kan, Nicky Langston, Kirstin Pirie, Alison Price, Gillian Reeves, Keith Shaw, Emma Sherman, Rachel Simpson, Helena Strange, Sian Sweetland, Ruth Travis, Lyndsey Trickett, Anthony Webster, Clare Wotton, Lucy Wright, Owen Yang, Heather Young, Emily Banks, Lucy Carpenter, Carol Dezateux, Julietta Patnick, Richard Peto, Cathie Sudlow

**Affiliations:** 1 Cancer Epidemiology Unit, Nuffield Department of Population Health, University of Oxford, Richard Doll Building, Old Road Campus, Oxford, OX3 7LF UK; 2 National Institute for Health Innovation, School of Public Health, University of Auckland, Level 4, Tamaki Campus, 261 Morrin Road, Glen Innes, Auckland, 1072 New Zealand

**Keywords:** Breast milk, Infant feeding, Breast cancer, Colorectal cancer

## Abstract

There are known short-term benefits in breastfed infants versus bottle-fed infants in terms of lower risks of infection and obesity in infancy and childhood, but the long-term effect on the risk of adult cancers is unclear. In a cohort of 1 in 4 UK women born in 1935–1950 we report the incidence of adult cancers in relation to having been breastfed in infancy. In median year 2001 (interquartile range 2000–2003) 548,741 women without prior cancer reported whether they had been breastfed. There was 81% agreement between women’s report of having been breastfed and information on breastfeeding recorded when they were 2 years old. Participants were followed by record-linkage to national cancer registration, hospital admission and death databases. Cox regression yielded adjusted relative risks (RRs) and 95% confidence intervals (CI) by having been breastfed or not for eight cancer sites with > 2000 incident cases and for related conditions, where appropriate. Of the eight cancers examined here one association was highly statistically significant: an increase in colorectal cancer incidence among women who had been breastfed versus not (RR 1.18, 95% CI 1.12–1.24, n = 8651). To investigate further the findings for colorectal cancer, we studied eight other gastro-intestinal conditions, and found increased risks in women who had been breastfed versus not for benign colorectal polyps (RR 1.09, 95% CI 1.05–1.13, n = 17,677) and for appendicitis (RR 1.19, 95% CI 1.07–1.31, n = 2108). The greater risks of adult colorectal cancer, colorectal polyps and appendicitis associated with having been breastfed in infancy suggest possible long-term effects of infant feeding practices on the gastrointestinal tract. Further studies are required to clarify this novel association.

## Introduction

There are known short-term benefits in breastfed infants versus bottle-fed infants in terms of lower risks of infection and obesity in infancy and childhood [1], and some evidence has also suggested a reduced long-term risk of obesity and diabetes in adulthood [2]. Evidence on the long-term effects on other aspects of adult health, and particularly on the risk of adult cancer, is limited. There is a long-standing hypothesis, dating from the discovery of the mouse mammary tumour retrovirus [3], that there might be similar vertically transmitted carcinogenic retroviruses in human breast milk [4–6]. A 2005 meta-analysis did not, however, find a significant difference in breast cancer risk by having been breastfed, but most of the evidence was from retrospective studies, which might have been affected by recall bias [7]. Under the viral hypotheses, maternal breast cancer might be expected to be a marker of maternal viral carriage, but only three small retrospective studies investigated the risk of breast cancer associated with having being breastfed in women whose mothers had had breast cancer, and findings are inconsistent [8–10]. There are other known differences between breastfed infants and bottle-fed infants in terms of growth [11], gut microorganisms [12], and the immune system [13] and some effects are thought to persist into adulthood [7]. In a large prospective study of UK women we compare the incidence of eight common adult cancers in those who reported that they were and were not breastfed as infants.

## Methods

In 1996–2001 about 1 in 4 UK women born in 1935–1950 was recruited into the prospective Million Women Study [14]. At recruitment, participants completed a questionnaire and reported personal information, including whether their mother had had breast cancer. Three years after recruitment participants were resurveyed and asked to update their personal and health characteristics and were asked for the first time about their usual diet [15] and about various childhood characteristics including their birthweight and whether or not they had been breastfed in infancy [16]. The 3-year resurvey was the baseline for these analyses. Study participants were followed for incident cancer, hospital admissions and deaths through record-linkage to routinely collected National Health Service (NHS) cancer registers and hospital admissions (NHS Digital in England and in NHS Information Services Division in Scotland). Diagnoses were coded to the International Classification of Diseases, 10th Revision (ICD-10). The study was approved by the Multi-Centre Research Ethics Committee for Anglia and Oxford. Data access policy and other information can be found on the study website (http://www.millionwomenstudy.org).

A sample of Million Women Study participants had, at the time of their birth in March 1946, been included in a UK-representative birth cohort study (the National Health Survey of Health and Development) [17]. When they were 2 years old it was recorded whether or not they were breastfed for more than a month. A comparison of this information with that reported by the same women on the Million Women Study questionnaire showed 81% agreement among 268 women [17]. Among those breastfed, the average duration of breastfeeding recorded when they were 2 years old was 6 months [17].

Among women completed the baseline resurvey questionnaire for these analyses, we excluded 17,970 women with prior cancer and 224,259 who reported that they did not know whether or not they had been breastfed. The remaining 548,741 women were followed from baseline to the date of death, any cancer, lost to follow up, or 31 December, 2015, whichever was earliest. Using Cox regression, we estimated adjusted hazard ratios [referred to as relative risks (RRs) hereafter] and confidence intervals (CIs) for cancer and for selected other conditions in women who reported having been breastfed versus not. All analyses were stratified by single year of birth and single year at baseline, and using time since baseline as the underlying time variable. Unless otherwise specified, RRs were also adjusted for area deprivation [18] (in quintiles), having an educational qualification or not, 10 regions of residence, adult height (< 165, 165–169, ≥ 170 cm), body mass index (< 25, 25–29, 30 + kg/m^2^), smoking (never, past smokers, current smokers who smoked < 10, 10–19, and ≥ 20 cigarettes per day), strenuous exercise (never, once per week, more than once per week), and alcohol consumption (0, 1–3, 4–6, 7–14, 15 + drinks/week) [19], total energy intake (in quintiles), daily fibre intake (in quintiles [15]), meat consumption (none, poultry only, red meat with no or little processed meat, and red meat plus processed meat), age at menarche (< 12, 12–14, 15 + years), parity (0, 1, 2, 3 +) and the age when their first child was born (< 24 and 24 +), and use of hormonal therapy for menopause (yes, no). For some analyses we additionally adjusted for other characteristics at birth and childhood including birth weight (< 2.5, 2.5–2.9, 3.0–3.4, 3.5–3.9, 4.0 + kg), parental smoking when they were born (both parents, one parent, and none), and whether parents owned their home when they were 10 years old. Women with missing values for any of the adjustment variables (< 4% for each variable) were assigned to separate categories for that variable, except for separate analyses with adjustment for characteristics at birth and childhood, in which only women who provided the information were included. Nominal significance was defined as *p* < 0.01, but confidence intervals of relative risks were all listed so that the strength of statistical significance can be assessed.

We examined associations for cancer at eight sites with 2000 or more incident cases. For cancer of the corpus uterus, we excluded women who reported that they had prior hysterectomy. For ovarian cancer, we excluded women who reported that they had prior bilateral oophorectomy. With a possible association found for colorectal cancer, we did a sensitivity analysis censoring women after they were first invited for bowel cancer screening (to avoid possible bias by selective participation in the national bowel screening programme) using information obtained through linkage to the National Health Service Bowel Cancer Screening Programme in England (NHSBCSP) [20]. To seek evidence of plausible mechanisms that may explain the association with this gastrointestinal system cancer, we further investigated associations for eight other gastro-intestinal conditions. Likelihood ratio test was used for heterogeneity across subgroups by each characteristic.

### Role of the funding source

The Million Women Study is funded by Medical Research Council UK and Cancer Research UK. The funders played no role in the collection, analysis, and interpretation of data; in the writing of the report; and in the decision to submit the paper for publication.

## Results

Overall 72% (395,363/548,741) of eligible women without prior cancer reported that they had been breastfed as babies. Table 1 compares certain characteristics of women who had and had not been breastfed. The proportion who had been breastfed as infants declined by year of birth and otherwise there were no major differences between women who had and had not been breastfed as infants.

**Table 1:** Characteristics of women who reported that they had and had not been breastfed as infants

	Were you breast-fed when you were a baby?	
	Yes	No
N	395,363	153,378
By year of birth		
Before 1939	76% (111,308)^a^	24% (36,056)^a^
1939–1945	72% (144,345)^a^	28% (55,054)^a^
After 1945	69% (139,710)^a^	31% (62,268)^a^
Age at start of follow up (mean and SD)	59.9 (5.0)	59.3 (4.7)
Participant characteristics at birth and childhood		
Birth weight, mean in kg (SD)	3.3 (0.7)	3.1 (0.8)
Either parent smoked when born, % (n)	85% (272,615)	88% (108,911)
Parents’ owned their home, % (n)	41% (99,918)	39% (34,645)
Hormonal factors		
Age at menarche, mean (SD)	12.9 (1.6)	12.9 (1.6)
Parous, % (n)	89% (349,805)	89% (136,443)
Age at first birth, among parous, mean (SD)	24.3 (4.3)	24.1 (4.3)
Ever used hormones for menopause, % (n)	54% (209,258)	55% (82,278)
Other social and lifestyle factors		
Area deprivation, in highest fifth, % (n)	18% (72,653)	21% (31,972)
Any educational qualification, % (n)	68% (262,384)	62% (93,144)
Adult height, mean in cm (SD)	162.7 (6.5)	162.0 (6.6)
Body mass index, mean in kg/m-2 (SD)	26.1 (4.6)	26.3 (4.8)
Current smoker, % (n)	11% (43,261)	13% (19,399)
Strenuous exercise ≥ once a week, % (n)	45% (172,429)	41% (60,891)
Alcohol consumption, % > 7 drinks/week (n)	28% (109,539)	27% (40,865)
Energy intake, mean Kcal (SD)	1832 (485)	1793 (495)
Fibre intake, mean g/week (SD)	99 (33)	95 (33)
Consumed red or processed meat, % (n)	65% (253,308)	65% (98,677)

Information in the above table was recorded at recruitment or at the 3-year resurvey, except for home ownership, which was recorded at the 12-year resurvey; the percentages are calculated among women who provided valid information

^a^Percentages are proportions of the same row

**Fig. 1:**
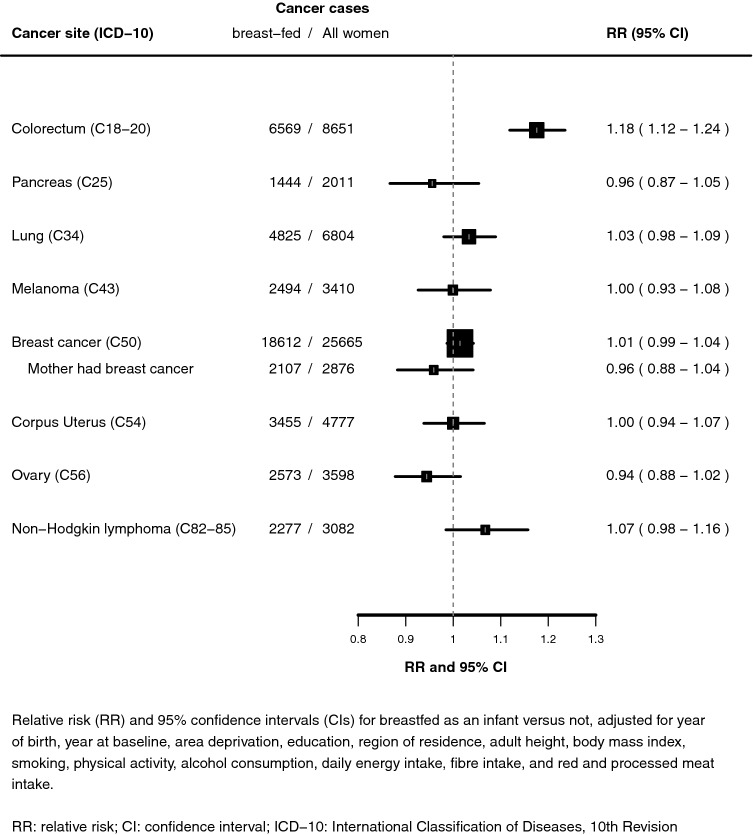
Relative risk for specific cancers in women who reported they were breastfed versus not as infants

During 9.8 million person-years (12.7 years per woman) of follow-up, 57,998 incident cancers were registered across the eight most common cancer sites. After stratification by single year of birth and single year at baseline and adjustment for 14 additional factors, women who had been breastfed had a significantly higher risk of colorectal cancer (8651 incident cases, adjusted RR 1.18, 95% CI 1.12–1.24, *p *< 0.0000001; Fig. 1), but for the other 7 cancer sites there was no difference in risk by whether or not women had been breastfed (*p *> 0.05). For breast cancer there was no association overall (25,665 cases, RR 1.01, 95% CI 0.99–1.04) or when analyses were restricted to women whose mother had had breast cancer (2876 cases, RR 0.96, 95% CI 0.88–1.04).

**Fig. 2:**
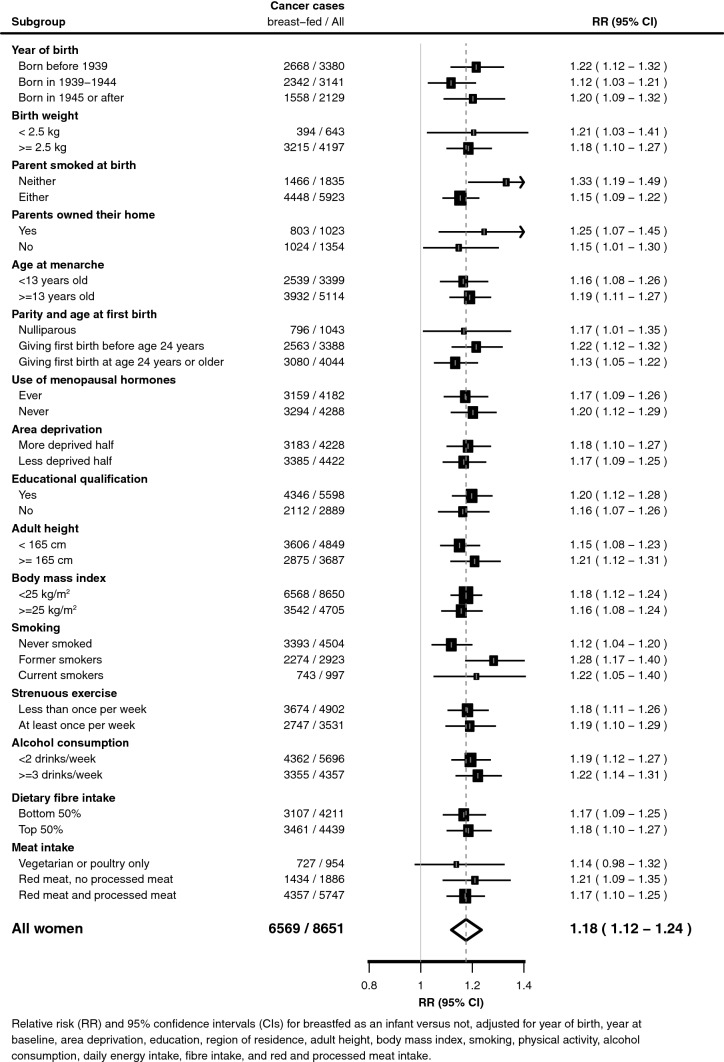
Relative risk of colorectal cancer in women who reported they were breastfed versus not as infants: subgroups

The excess risk of colorectal cancer did not vary significantly by subgroups of women defined by 16 characteristics (Fig. 2, *p* > = 0.05), nor when women who had been invited for bowel cancer screening were excluded from the analysis (RR 1.17, 95% CI 1.09–1.26, n = 3903). The risk was significantly elevated separately both for colon cancer and for rectal cancer (Table 2). The excess risk observed for colorectal cancer in those breastfed, was also little affected by adjustment for the 17 factors including dietary intakes of energy, fibre and meat (Table 3).

**Table 2:** Relative risks (RRs) and 95% confidence intervals (95% CI) for selected gastrointestinal conditions in women who reported they were breastfed versus not as infants

Conditions (ICD-10 codes)	Number of cases in	Adjusted for year of birth, year at baseline, area deprivation, education and region of residence	Additionally adjusted for 8 factors^a^
	Breastfed/all women	RR (95% CI)	RR (95% CI)^b^
Colorectal cancer			
Colorectal cancer (C18–C20)	6569/8651	1.17 (1.12–1.23)	1.18 (1.12–1.24)
Colon cancer (C18–C19)	5104/6702	1.19 (1.12–1.25)	1.19 (1.12–1.26)
Rectum cancer (C20)	1465/1949	1.14 (1.02–1.26)	1.14 (1.03–1.26)
Eight other gastrointestinal conditions			
Oesophageal cancer (C15)	735/1055	0.87 (0.76–0.99)	0.88 (0.77–1.01)
Stomach cancer (C16)	630/848	1.11 (0.95–1.30)	1.13 (0.97–1.33)
Peptic ulcer (K25–K27)	6583/9219	0.97 (0.92–1.01)	0.99 (0.95–1.04)
Cancer of the small intestine (C17)	178/266	0.78 (0.60–1.01)	0.78 (0.60–1.01)
Inflammatory bowel diseases (K50, K51)^b^	2946/4211	0.91 (0.85–0.97)	0.93 (0.87–0.99)
Appendicitis (K35)	1586/2108	1.18 (1.07–1.30)	1.19 (1.07–1.31)
Colorectal polyps (K62.1, K63.5)	13,016/17,677	1.08 (1.04–1.11)	1.09 (1.05–1.13)
Diverticular disease (K57)	36,245/50,059	0.99 (0.97–1.01)	1.01 (0.99–1.03)

^a^Additionally adjusted for adult height, body mass index, smoking, physical activity, alcohol consumption, daily energy intake, fibre intake, and red and processed meat intake

^b^Inflammatory bowel diseases include Crohn’s disease (K50) and ulcerative colitis (K51)

**Table 3:** Relative risk for colorectal cancer in women who reported they were breastfed versus not as infants: effect of adjustment

Adjustment model	RR (95% CI)
Adjusted for year of birth and year at baseline only	1.18 (1.12–1.23)
Additionally adjusted separately for…	
Participant characteristics at birth and childhood	
Birth weight	1.18 (1.11–1.25)
Parents smoked at birth	1.19 (1.12–1.25)
Parents’ owned their home	1.22 (1.11–1.35)
Hormonal factors	
Age at menarche	1.18 (1.12–1.24)
Parity and age at first birth	1.17 (1.12–1.23)
Ever used hormones for menopause	1.18 (1.12–1.24)
Other social and lifestyle factors	
Region	1.18 (1.12–1.24)
Area deprivation	1.18 (1.12–1.23)
Any educational qualification	1.17 (1.12–1.23)
Adult height	1.17 (1.11–1.23)
Body mass index	1.18 (1.12–1.24)
Smoking	1.18 (1.12–1.24)
Strenuous exercise	1.18 (1.12–1.24)
Alcohol consumption	1.17 (1.12–1.23)
Energy intake	1.17 (1.12–1.23)
Fibre intake	1.18 (1.12–1.24)
Consumed red or processed meat	1.18 (1.12–1.24)
Adjusted for all characteristics above	1.18 (1.12–1.24)

The excess of colorectal cancer did not appear to be confounded by other known factors and was unlikely to be due to chance, and we therefore examined hospital admissions for eight other common gastro-intestinal conditions, to see whether any of them might also be associated with having been breastfed. All analyses censored women at the date of diagnosis of colorectal or any other cancer. No significant associations were found for cancers of the oesophagus, stomach, or small intestine (Table 2). However, women who had been breastfed were more likely to have had benign colorectal polyps (RR 1.09, 95% CI 1.05–1.13, n = 17,677; *p *< 0.0000001) and appendicitis (RR 1.19, 95% CI 1.07–1.31, n = 2108; *p* = 0.0008) but less likely to have had inflammatory bowel disease (RR 0.93, 95% CI 0.87–0.99, n = 4211; *p* = 0.03). These associations with risk of benign colorectal polyps, appendicitis, and inflammatory bowel disease were not sensitive to adjustment by potential confounding factors (Table 2).

## Discussion

Breastfed infants are known to have many short-term health benefits compared to bottle-fed infants [1] and it is thought that there might also be long-term effects extending into adulthood [7, 21] This is by far the largest study to date of adult cancer risk associated with having been breastfed as an infant. We found a highly significant excess risk of colorectal cancer (with 8651 incident cases) among women who had been breastfed, and no association for seven other cancer sites with at least 2000 incident cancers. We also found highly significant excess risks of benign colorectal polyps and of appendicitis associated with having been breastfed in infancy.

Only one previous study has reported on the association with colorectal cancer, and with 53 incident cases found no significant association [7]. In our analysis, the excess risks of colorectal cancer and of colorectal polyps were little affected by adjustment for possible confounding factors, including smoking, body mass index, and dietary intakes of fibre and processed meat. The excess risks for colorectal cancer and for colorectal polyps, while unexpected, were both highly statistically significant. They suggest possible long-term differences in the lower gastro-intestinal tracts of adults who were breastfed and bottle-fed as infants. Others have hypothesised that the gut microbiome or virome might affect colorectal cancer risk [22, 23]. Infants who were and were not breastfed have been reported to have different gut microbiota [12]. Some evidence suggests that gut microbiota mature at around age 3 years [24], but the extent to which different infant microbiota translate into long-term health is unclear.

Carcinogenic viruses can be transmitted vertically through breast milk [3–6]. Cow’s milk was the main alternative source of milk for infants in the 1930s and 1940s when almost all women in this cohort were born, and the ingredients of infant formula were not regulated in the UK until the 1960s [25]. Meta-analysis of observational data have reported that the consumption of cow’s milk in adulthood is associated with a reduced risk of colorectal cancer in many studies [26], but the relevance of this for our finding here for breastfed versus bottle-fed infants is unclear.

We found, as others have reported [7, 9, 27–31], no overall difference in breast cancer risk in those having been breast-fed as infants versus not. The mouse mammary tumour retrovirus is transmitted through breast milk [3, 4], and if a mammary tumour virus were also to be transmitted vertically by human carriers, a sensitive test for such transmission is to examine the association among women whose mother had had breast cancer and hence could have been a virus carrier. This is also the largest and the only prospective report of familial cases of breast cancer, but still no excess risk was identified, strongly suggesting that transmission of a mammary tumour virus in milk is an unlikely an important cause of human breast cancer.

In this prospective study of UK women born in 1935–1950, 72% of reported they were breastfed as infants, consistent with findings from the 1946 birth cohort study (National Survey of Health and Development) [32] and the Hertfordshire Birth Cohort of babies born in 1930s [31]. The validation study showed 81% agreement between information on having been breastfed reported in the Million Women Study, when women were in middle age, and that recorded for the same women when they were 2 years old in the 1946 birth cohort study [17]. Any misclassification of women would tend to attenuate associations with cancer and other conditions.

Strengths of this study include its large size, virtually complete follow-up through routinely collected NHS databases, and ability to adjust for many potential confounding factors, including smoking, body mass index, and diet. The prospective design minimises possible recall bias between those with and without incident cancer, but as in other observational studies the possibility of confounding by unmeasured or unknown factors, for example childhood diet characteristics, cannot be excluded. Due to the age restrictions in recruitment and the exclusion of individuals who had incident cancer or died prior to study baseline, as required by the prospective design, these findings cannot be generalised to younger ages or extrapolated to lifetime cancer risks. Selection bias may arise if we have not controlled for other determinants of selection into the study that are also determinants of colorectal cancer risk. To explain our findings, any such bias would have to be due to the equivalent of an unknown factor that is an important, independent cause of colorectal cancer, but not of other cancers, which is also a cause of selection into the study but is essentially uncorrelated with the other factors for which we have controlled. This seems unlikely but again cannot be categorically ruled out. We were unable to investigate associations with the length of breastfeeding or with other infant feeding practices (such as exclusive breastfeeding) in this cohort. It is also unclear if these findings could be generalised to other populations where infant feeding practices differ.

There are definite and important benefits of being breast-fed, which would far outweigh any increased risk of colorectal cancer and colorectal polyps in adulthood. Nevertheless the evidence presented here suggests possible long-term effects of infant feeding practices on the risk both of colorectal cancer and of colorectal polyps. This could imply novel mechanisms of colorectal carcinogenesis, but further studies are required to reproduce and to clarify this novel association.
